# Stage-specific transcriptional atlas of goose satellite cells uncovers sex-biased molecular dynamics associated with embryonic skeletal muscle development

**DOI:** 10.3389/fcell.2026.1957937

**Published:** 2026-10-02

**Authors:** Cui Wang, Kai Shi, Yi Liu, Yongping Yang, Guitao Jiang, Chuang Li, Shufang Chen, Huiying Wang, Daqian He

**Affiliations:** 1 Institute of Animal Science and Veterinary Medicine, Shanghai Academy of Agricultural Sciences, Shanghai, China; 2 Shanghai Municipal Key Laboratory of Agri-Genetics and Breeding, Shanghai, China; 3 Ningbo Academy of Agricultural Sciences, Ningbo, China; 4 Wuhan Animal Disease Control Center, Wuhan, China; 5 Yuelushan Laboratory, Changsha, Hunan, China; 6 Hunan Institute of Animal and Veterinary Science, Changsha, Hunan, China

**Keywords:** skeletal muscle satellite cells, sexual dimorphism, embryonic skeletal muscle development, stage-specific transcriptomic atlas, goose

## Abstract

**Introduction:**

Sexual dimorphism in avian skeletal muscle is established during embryogenesis, but the stage-resolved transcriptional programs governing this process in geese remain largely unexplored.

**Methods:**

To construct a stage-specific transcriptional atlas of muscle satellite cells (SMSCs), we performed histological examination and RNA sequencing of PAX7^+^ SMSCs isolated from male and female Zhedong White goose embryos across four developmental stages (E13, E15, E18, and E23). Quantitative PCR was used to validate selected differentially expressed genes (DEGs).

**Results:**

Although myofiber morphology exhibited no overt sexual divergence, immunofluorescence revealed distinct sex- and stage-dependent dynamics, with male embryos displaying a higher PAX7^+^ cell abundance at E18 and females at E23. Transcriptomic profiling identified 357, 261, 312, and 672 DEGs at E13, E15, E18, and E23, respectively, uncovering a progressively intensifying sexual bias. At E13, female-biased DEGs were enriched for positional identity genes (HOXC9/C11, HOXD12/13) and pro-proliferative factors (GDF7, FGF16), whereas male-biased DEGs included adhesion molecules (IGSF10/11). At E15, females upregulated genes linked to myofiber maturation (ACTA1, MYH7B) and lipid metabolism (PLIN1, ADIPOQ), while males showed enrichment in extracellular matrix remodeling (MMP7, COL17A1) and immune-related pathways. At E18, females enhanced neuroendocrine signaling (ADCYAP1, AGTR2), whereas males upregulated contractile apparatus components (RYR1, CASQ1) and testis-associated transcripts (BRDT, DHH). By E23, females exhibited signatures of metabolic and neuromuscular optimization, whereas males showed enrichment in extracellular matrix genes (COL11A2, COL9A1/2) and masculinization-associated factors (YBX2, GGN). Temporal pathway analysis demonstrated a progression from immune/chemokine processes at E13, through cytoskeletal organization at E15 and supramolecular fiber components at E18, to DNA metabolism coupled with chemokine signaling at E23. Protein-protein interaction network analysis pinpointed stage-specific hub genes: EGFR (E13), ACTA1/ADIPOQ (E15), RAC2 (E18), and GNG10 (E23). Quantitative PCR validated eight selected DEGs.

**Discussion:**

Collectively, this transcriptional atlas delineates sex-biased molecular dynamics that operate within the Zhedong White goose SMSC pool in the absence of conspicuous histological differences, and provides a foundational gene resource for prioritizing mechanistic investigations into sexually dimorphic muscle development, focusing specifically on the embryonic leg muscles.

## 1 Introduction

Skeletal muscle satellite cells (SMSCs) are a population of myogenic stem cells located between the basal lamina and the sarcolemma of muscle fibers, playing a fundamental role in postnatal muscle growth, regeneration, and repair. These cells possess the essential capacities for both proliferation and differentiation (Mauro, 1961). Since the total number of muscle fibers is largely determined during embryogenesis, postnatal muscle development occurs primarily through the hypertrophy of existing fibers—a process critically mediated by SMSC activity (Moss and Leblond, 1971; Schultz, 1996). Consequently, the establishment and intrinsic properties of the embryonic SMSC pool are key determinants of ultimate muscle mass and meat quality (Rhoads, et al., 2009; Khodabukus, et al., 2018).

Sexual dimorphism in skeletal muscle, characterized by sex-based differences in muscle mass, fiber type composition, and growth dynamics, is a common feature in birds and mammals (Gous, et al., 2019). In poultry production, this dimorphism has direct implications for economically important traits such as growth rate and feed efficiency (Chu, et al., 2020). For instance, in broiler chickens, the divergence in body weight between males and females becomes increasingly pronounced with age. In organic Ross 308 broilers, males exhibited significantly higher final body weight, daily weight gain, and carcass weight than females, whereas females displayed significantly larger myofiber diameters, indicating sex-specific effects on both growth performance and muscle histology (Cygan-Szczegielniak and Bogucka, 2021). Earlier studies have also reported sex-related growth curve differences in other poultry species, including guinea fowl and Vietnamese Mia chickens (Nahashon et al., 2006; Nguyen Hoang et al., 2021). In black Muscovy ducks, Zhang et al. (2024) reported that females exhibited significantly larger myofiber diameters and lower myofiber densities than males in breast muscle at post-hatch day 42 (p < 0.05), with the sex difference widening by day 70 (p < 0.01); in contrast, no significant sexual divergence was observed at day 28.

Beyond histological observations, transcriptomic analyses have revealed that sex-specific molecular signatures underlie these phenotypic differences. In black Muscovy ducks, Zhang et al. (2024) identified key sex-dimorphic genes—including TPM2, HNRNPK, VCP, ATP2A2, and ANKRD1—in breast muscle. The differentially expressed genes were significantly enriched in pathways such as cGMP-PKG signaling, calcium signaling, and hypertrophic cardiomyopathy, which may account for the greater breast muscle mass observed in males (Zhang et al., 2024). Similarly, Bošković Cabrol et al. (2025) reported that male broilers exhibit more pronounced transcriptomic alterations in growth-related myopathies, characterized by significant disruption of pathways involved in hypoxia, energy metabolism, calcium signaling, and extracellular matrix remodeling, whereas females display comparatively muted transcriptional responses. Notably, in Muscovy ducks, male-biased growth has been linked to distinct breast muscle transcriptomic profiles enriched in pathways associated with energy metabolism and lipid synthesis (Tian et al., 2024). Moreover, our previous multi-tissue transcriptome study in geese further highlighted the systemic and coordinated influence of sex on growth physiology, revealing that male geese exhibit upregulated genes enriched in pathways related to nutrient digestion and absorption, while females show higher expression of genes involved in endocrine metabolism and hormone activity (Yang et al., 2024).

Despite these advances, it is important to note that most existing studies have focused on post-hatch or adult stages. Some previous work has begun to explore sexual dimorphism during embryonic myogenesis and satellite cell development in poultry, providing initial evidence that sex-biased molecular programs may emerge prior to hatching (e.g., in chicken and quail models). Nevertheless, a critical gap remains: the precise timing and mechanisms by which sexual dimorphism initially arises in progenitor cells such as SMSCs have yet to be systematically investigated across multiple embryonic stages. Given that muscle fiber number is determined embryonically and that SMSCs originate during this period, elucidating sex-specific transcriptional programs in embryonic SMSCs is essential for deciphering the developmental origins of muscle sexual dimorphism.

The Zhedong White (ZW) goose, a prominent meat-type breed, exhibits marked sexual dimorphism in growth performance, with males achieving greater adult body size and faster growth rates (Yang et al., 2024). To address the aforementioned gaps, in this study, we performed transcriptomic profiling of SMSCs from male and female goose embryos at four key stages (E13, E15, E18, and E23). To our knowledge, this represents the first multi-stage transcriptomic analysis of sex-biased transcriptional dynamics in goose embryonic SMSCs. Specifically, this study addresses two fundamental questions: (1) Do sex-specific transcriptional signatures emerge as early as embryogenesis in SMSCs? (2) Are classical sex differentiation pathways already activated within these myogenic precursors? By mapping the sex-specific transcriptional dynamics of embryonic SMSCs, this study aims to uncover the molecular foundations of skeletal muscle sexual dimorphism, potentially enabling the identification of early biomarkers for sex-biased breeding strategies in poultry.

## 2 Materials and methods

### 2.1 Animals, ethics, and samples collection

All animal experiments were approved by the Animal Care and Use Committee of the Shanghai Academy of Agricultural Sciences (Approval No: SAASPZ0526168) and conducted in accordance with its ethical guidelines. This research also complied with the ARRIVE guidelines (https://arriveguidelines.org) and all applicable regulations governing the ethical treatment of animals.

Fertilized ZW goose eggs (n = 100) were obtained from the Wenjie Goose Breeding Department of Xiangshan Co. Ltd. China, and incubated under standard conditions. Embryos were collected at four developmental stages (E13, E15, E18, and E23) for further analysis. Embryonic sex was determined by PCR amplification of the *CHD1* gene (Table 1). The ZW geese used in the study were euthanized via car-bon dioxide inhalation followed by cervical dislocation, performed by trained personnel. Leg muscle tissues were collected in full compliance with animal welfare regulations and were harvested separately from female and male embryos for subsequent analyses. For histological examination, samples (n = 3 per stage per sex) were fixed in a glutaraldehyde-based muscle fixative (ServiceBio, China). SMSCs were isolated from fresh tissues obtained from a separate cohort of embryos (n = 3 per stage per sex). Detailed information regarding animal ethics, egg incubation, sex determination, and tissue collection has been described in our previous study (Wang et al., 2026b).

**TABLE 1:** Primers used in this study.

Purpose	Gene name	Primer sequence (5’→3′)	Product size (bp)	Accession number
Sex test	CHD1	F: TGCAGAAGCAATATTACAAG R: AATTCATTATCATCTGGTGG	466/326	NC_089912.1
Internal control	β-actin	F: TCCGTGACATCAAGGAGAAG R: CATGATGGAGTTGAAGGTGG	224	M26111.1
qRT-PCR validation	FGF16	F: CGCCCACCGACTTCGCTCACC R: ATACAGCTCGCCTCTCTCGTT	227	XM_048070704.2
	IGSF10	F: AGGCGTTCACTGACTTGCATT R: CAAGGTGACAAAAGTATCTGG	233	XM_048059482
	ACTA1	F: CAC​GGC​ATC​ATC​ACA​AAC​TGG	135	XM_048047270
		R: CTC​ACG​GTT​GGC​TTT​GGG​GTT	​	
	MMP7	F: ATGACATTGATGAGGAAGAAG R: TTATCATCTACAGGAATCCCA	205	XM_066991566.1
	GATA6	F: GTTCCCCGACCCCTTGCTATG R: TTGTAGAAGACGTGGATGTTG	154	XM_066992850.1
	BRDT	F: CTCCTCCACCAGAATATATAA R: CTCAGGTCCATAGGTTTCTTT	190	XM_013186705.3
	ANK1	F: AGAAAAAGAGAGGGCATGCAC R: ATCTTGCAGCTCTCAGGAAAC	138	XM_048047271.2
	YBX2	F: GTGGTTCAACGTGCGCTGTGG R: ACGATGTCGAACTCGACGGTC	150	XM_066985365.1

### 2.2 Histological and immunofluorescence analysis of leg muscle tissues

Paraffin-embedded leg muscle sections (2 μm) obtained from male and female ZW goose embryos at E13, E15, E18, and E23 were used for histological and immunofluorescence analyses. Hematoxylin and eosin (HE) staining was performed to evaluate muscle morphology. For immunofluorescence staining, sections were incubated with an anti-Pax7 antibody (Abcam, Cambridge, United Kingdom, cat. no. ab187339, Wang et al., 2026b) to label satellite cells, followed by DAPI counterstaining for nuclear visualization. Images were captured using a NanoZoomer scanner (Hamamatsu, Sydney, Australia) and an OLYMPUS fluorescence microscope. The proportion of Pax7-positive cells was quantified using Aipathwell v2 software (Servicebio, Wuhan, China). All image acquisition and cell-counting analyses were performed by an investigator blinded to sample sex and embryonic stage to minimize observer bias. Detailed procedures for tissue fixation, dehydration, sectioning, staining, and image acquisition have been described previously (Wang et al., 2026b).

### 2.3 Isolation and purification of goose embryonic SMSCs

Primary SMSCs were isolated from leg muscle tissues of male and female ZW goose embryos at E13, E15, E18, and E23. Following aseptic dissection, tissues were minced and enzymatically digested with Dispase II (Roche, Basel, Switzerland) and Collagenase II (Gibco, United States). SMSCs were enriched through differential plating, and their purity was confirmed by immunofluorescence staining for the satellite cell marker Pax7, as described in our previous study (Liu et al., 2025; Wang et al., 2026a; Wang et al., 2026b). Purified SMSCs were harvested in TRIzol reagent (Invitrogen, United States) and stored at −80 °C until RNA extraction and transcriptome sequencing.

### 2.4 RNA extraction, library preparation and transcriptome sequencing

Total RNA was extracted from purified SMSCs using Trizol Reagent (Invitrogen, United States). RNA quality and concentration were assessed using a NanoDrop NC2000 spectrophotometer (Thermo Fisher Scientific, United States) and agarose gel electrophoresis. cDNA libraries were constructed using the NEBNext Ultra II RNA Library Prep Kit for Illumina (New England Biolabs Inc., United States). Library quality was evaluated on an Agilent 2100 Bioanalyzer (Agilent, United States), and quantified by real-time PCR. Qualified libraries were sequenced on an Illumina NovaSeq 6000 platform (Personal Biotech, China), generating paired-end reads. Detailed procedures for RNA quality control, library preparation, and sequencing have been described previously (Wang et al., 2026b).

### 2.5 RNA-seq data quality assessment and transcriptome analysis

To elucidate the cellular origins and early molecular drivers of muscle sexual dimorphism, transcriptomic profiling was performed on SMSCs isolated from the leg muscles of male and female goose embryos at four developmental stages (E13, E15, E18, and E23). The identity of the isolated cells as goose SMSCs was confirmed by immunofluorescence staining for PAX7, as previously described (Wang et al., 2026b).

A total of 24 RNA-seq libraries (three biological replicates per sex per stage) were constructed and sequenced. Raw sequencing reads were processed using fastp (v0.22.0) to remove adapter sequences and filter out low-quality reads—specifically, those containing more than 10% ambiguous N bases or with more than 50% of bases having a quality score below 5 (Chen et al., 2018). The remaining clean reads were aligned to the goose reference genome (Assembly GCF_002166845.1) using HISAT2 (v2.1.0) (Kim et al., 2019). Gene expression levels were quantified using HTSeq (v0.9.1) and normalized to fragments per kilobase of transcript per million mapped reads (FPKM) (Putri et al., 2022; Trapnell et al., 2010).

Quality control metrics for the sequencing data are summarized in Table 2. In brief, a total of 1.27 billion raw reads were generated, yielding an average of 5.49 Gb of high-quality data per sample. The Q20 and Q30 scores exceeded 98.69% and 94.07%, respectively, and GC content ranged from 49.02% to 53.69%. After filtering, 1.26 billion clean reads were retained, with an average of 52.54 million reads per sample. The clean reads were mapped to the reference genome (Assembly GCF_002166845) with mapping rates ranging from 85.85% to 88.34% (Table 2).

**TABLE 2:** The situation of data output quality and the mapped reads information.

Sample	Raw reads	Clean reads	Mapped read (mapping percentage)	Q20(%)	Q30(%)	GC content(%)
E13M-1	48411648	7310158848	42227846(88.05%)	99.04	95.76	49.02
E13M-2	52173334	7878173434	45479197(87.98%)	99.01	95.61	49.51
E13M-3	44503178	6719979878	38906351(88.19%)	99.05	95.74	49.17
E13F-1	47451864	7165231464	41112506(87.52%)	99.02	95.67	49.61
E13F-2	45809246	6917196146	39560448(87.21%)	99	95.58	51.33
E13F-3	47691802	7201462102	41621652(87.99%)	99.11	95.88	50.61
E15M-1	54114332	53564682	46558092(86.92%)	98.85	94.76	51.46
E15M-2	54083356	53526592	46637317(87.13%)	98.78	94.43	53.25
E15M-3	55564338	55171316	48734253(88.33%)	99.08	95.53	49.63
E15F-1	68135580	67572638	59281437(87.73%)	98.99	95.24	51.07
E15F-2	57268344	56676608	49303334(86.99%)	98.78	94.48	53.13
E15F-3	57338788	56719120	49287006(86.9%)	98.77	94.43	53.69
E18M-1	50979112	50400408	43268649(85.85%)	98.74	94.36	52.73
E18M-2	55175098	54556652	47022386(86.19%)	98.69	94.07	52.77
E18M-3	50294506	49755766	42876559(86.17%)	98.8	94.56	51.82
E18F-1	54466682	53916088	46833444(86.86%)	98.81	94.57	51.22
E18F-2	56428896	55843990	48376779(86.63%)	98.85	94.78	51.16
E18F-3	52730520	52208942	45300997(86.77%)	98.83	94.66	51.38
E23M-1	54756672	54347552	48010041(88.34%)	99.02	95.27	50.84
E23M-2	55073576	54475978	47569771(87.32%)	98.73	94.19	53.24
E23M-3	57871024	57287126	49939337(87.17%)	98.8	94.53	51.86
E23F-1	56559496	55989110	48788764(87.14%)	98.83	94.69	51.22
E23F-2	48122558	47659026	41535903(87.15%)	98.84	94.66	51.49
E23F-3	48378876	47897882	41863719(87.4%)	98.87	94.82	50.66

Differential expression analysis between developmental stages and between sexes was performed using the DESeq2 R package (v1.38.3) (Love, et al., 2014). Genes with |log_2_ Fold Change| > 1 and an adjusted P-value <0.05 were considered differentially expressed genes (DEGs). Hierarchical clustering of DEGs was performed using the Pheatmap R package (v1.0.12) to visualized expression patterns across samples.

Functional enrichment analysis was performed to elucidate the biological functions of DEGs. Gene Ontology (GO) term enrichment was analyzed using topGO (v2.50.0), and Kyoto Encyclopedia of Genes and Genomes (KEGG) pathway enrichment was conducted using clusterProfiler (v4.6.0) (Halevy, 2020). Enriched terms and pathways with an adjusted p-value <0.05 were considered statistically significant.

### 2.6 Protein-protein interaction network analysis

Protein-protein interaction (PPI) networks were established by querying differentially expressed genes against the STRING database (Franceschini et al., 2013). For protein-coding genes lacking direct annotation in STRING, orthologous sequences were retrieved via BLAST (v2.2.28; Altschul et al., 1990). Only interactions with a combined confidence score ≥0.4 (medium confidence) were retained for further analysis. PPI networks were constructed based on the interaction partners of these orthologs and visualized with Cytoscape software (v3.8.0; Shannon et al., 2003).

### 2.7 Quantitative real-time PCR validation

Quantitative real-time PCR (qRT-PCR) was performed to validate the RNA-seq expression patterns. Eight DEGs were selected for analysis, and gene-specific primers were designed using Oligo 6.0 (Table 1). Reactions were conducted using TB Green Premix Ex Taq II (Takara, Japan) on a CFX384 Touch Real-Time PCR Detection System (Bio-Rad, United States), with β-actin as the internal reference. Relative expression levels were calculated using the 2^(–ΔΔCt)^ method. Melting curve analysis was performed to verify amplification specificity. Detailed protocols for qRT-PCR validation, including reaction conditions and data analysis, have been described previously (Wang et al., 2026b).

### 2.8 Statistical analysis

All experiments were performed with three biological replicates per group. Data were analyzed by one-way analysis of variance (ANOVA) using SPSS software (IBM Corp., United States). When a significant main effect was observed (p < 0.05), *post hoc* comparisons of group means were performed using Duncan’s multiple range test (Shao et al., 2022). A p-value <0.05 was considered statistically significant. Detailed statistical procedures, including model specifications and multiple comparison adjustments, have been described previously (Wang et al., 2026b).

## 3 Results

### 3.1 Histological assessment and immunofluorescence analysis of leg muscle development

Hematoxylin and eosin (HE) staining was performed on leg muscle tissues from male and female goose embryos at four developmental stages (E13, E15, E18, and E23) to evaluate structural changes during embryogenesis (Figure 1A). At E13, nascent muscle fibers were identifiable, exhibiting loosely organized bundles alongside abundant myoblasts and mesenchymal cells distributed irregularly. By E15, an increase number of muscle fiber was observed, accompanied by the emergence of primitive fascicular architecture. At E18, muscle fibers appeared more numerous, and connective tissue septa were distinguishable, partitioning the tissue into polygonal bundles of varying sizes, with fewer interstitial cells compared with earlier stages. At E23, muscle fibers displayed larger cross-sectional areas and greater morphological heterogeneity, suggesting ongoing proliferation and hypertrophic growth. Notably, comparable morphological trajectories were observed between sexes across all stages, with no discernible sex-related differences in tissue architecture (Figure 1A).

**FIGURE 1:**
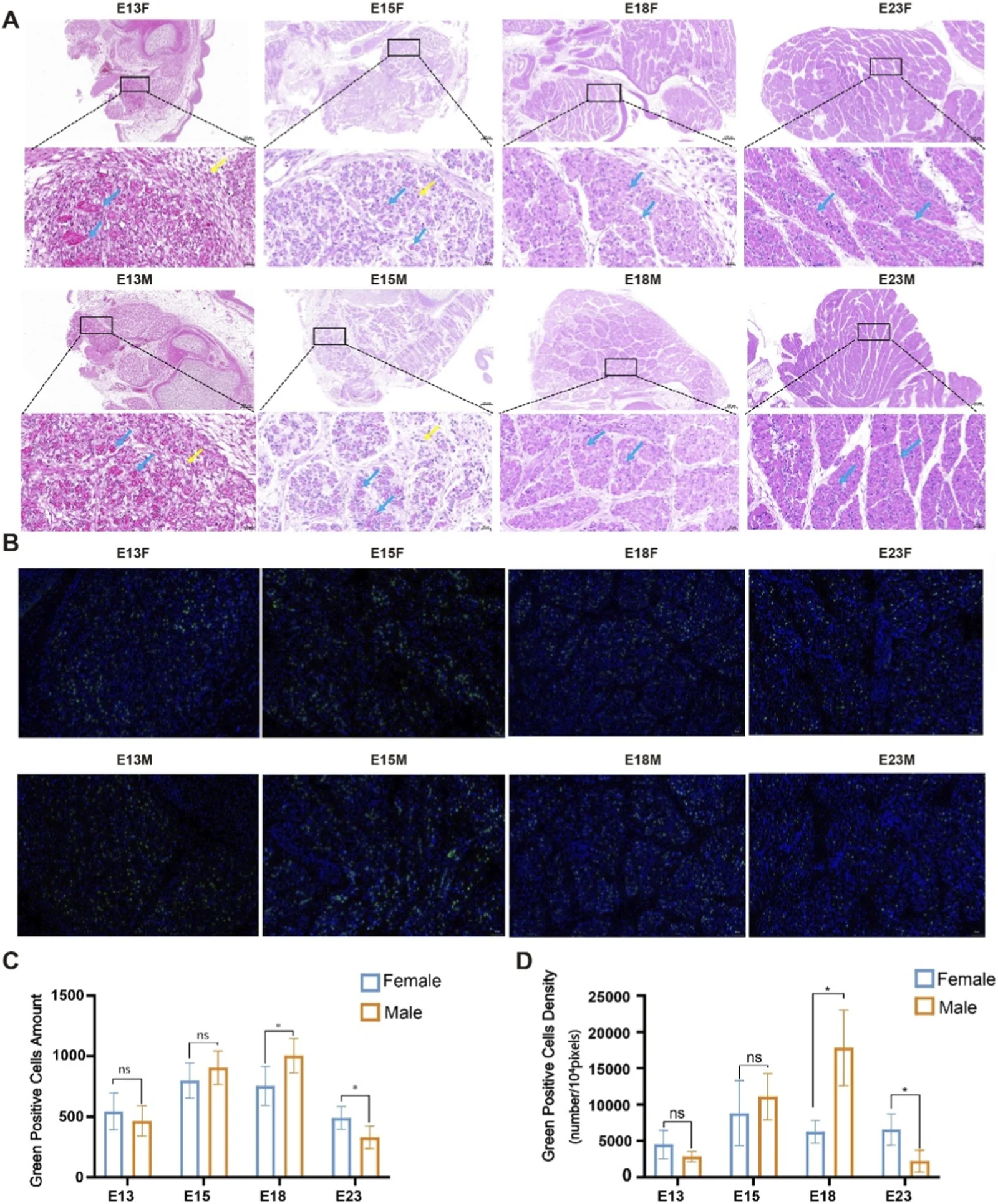
Histological assessment and immunofluorescence analysis of leg muscle development in ZW goose embryos. **(A)** Low-magnification (5×) and high-magnification (40×) H&E images of female (E13F–E23F) and male (E13M–E23M) leg muscle at embryonic days 13, 15, 18, and 23. Blue arrows indicate mature myofibers; yellow arrows indicate myoblasts/mesenchymal cells. Boxed regions are shown at higher magnification in the adjacent 40× panels. Scale bar = 100 µm. **(B)** Representative immunofluorescence images of Pax7 (green) with DAPI nuclear counterstaining (blue) in female (E13F–E23F) and male (E13M–E23M) leg muscle across the four stages. Scale bar = 100 µm. **(C)** Quantification of Pax7^+^ cell number. **(D)** Quantification of Pax7^+^ cell density (cells/mm^-2^). ns, not significant; *p < 0.05. Data are presented as mean ± SEM.

Immunofluorescence staining for PAX7 was conducted to identify SMSCs in the leg muscles of male and female goose embryos across four identical developmental stages (Figure 1B; Supplementary Table S1). As shown in Figures 1C,D, both the number and density of PAX7-positive cells exhibited dynamic changes from E13 to E23 in both sexes. At E13, PAX7^+^ cell counts were slightly higher in females than in males, although the difference was not statistically significant (p > 0.05). At E15, males showed a modest increase relative to females, again without significance (p > 0.05). By E18, males displayed a significantly greater number of PAX7^+^ cells than females (p < 0.05). However, at E23, this trend reversed, with females exhibiting a significantly higher number of PAX7^+^ cells than males (p < 0.05). A similar sex- and stage-dependent pattern was observed for PAX7^+^ cell density.

Collectively, despite comparable histological trajectories of leg muscle development between sexes, the PAX7^+^ satellite cell pool exhibits distinct sex- and stage-specific dynamics—characterized by a transient male-biased expansion at the mid-embryonic stage (E18), followed by a female-biased increase at the late embryonic stage (E23). These findings suggest that sex dimorphism in avian skeletal muscle development may be established at the cellular level within the satellite cell compartment.

### 3.2 Identification of DEGs between female and male samples across four developmental stages

As shown in Figure 2A, principal component analysis (PCA) of the embryonic skeletal muscle satellite cell transcriptomes revealed that PC1, accounting for 35.74% of the total variance, broadly segregated samples by developmental stage, with E18 and E23 samples clearly separated from E13 and E15 along this axis. However, E13 and E15 samples occupied adjacent and partially overlapping regions on PC1, suggesting a less distinct stage-related separation at earlier developmental time points. PC2, which explained 24.55% of the variance, appeared to contribute to sex-related discrimination, particularly within the E18 and E23 groups, where male and female clusters exhibited more pronounced divergence. By contrast, the E15 male and female samples were not clearly distinguished along PC2 and showed considerable overlap in their relative positions. Within each group, biological replicates (e.g., E13F) clustered tightly, reflecting low intra-group variability and supporting the consistency and technical robustness of the sequencing data (Figure 2B).

**FIGURE 2:**
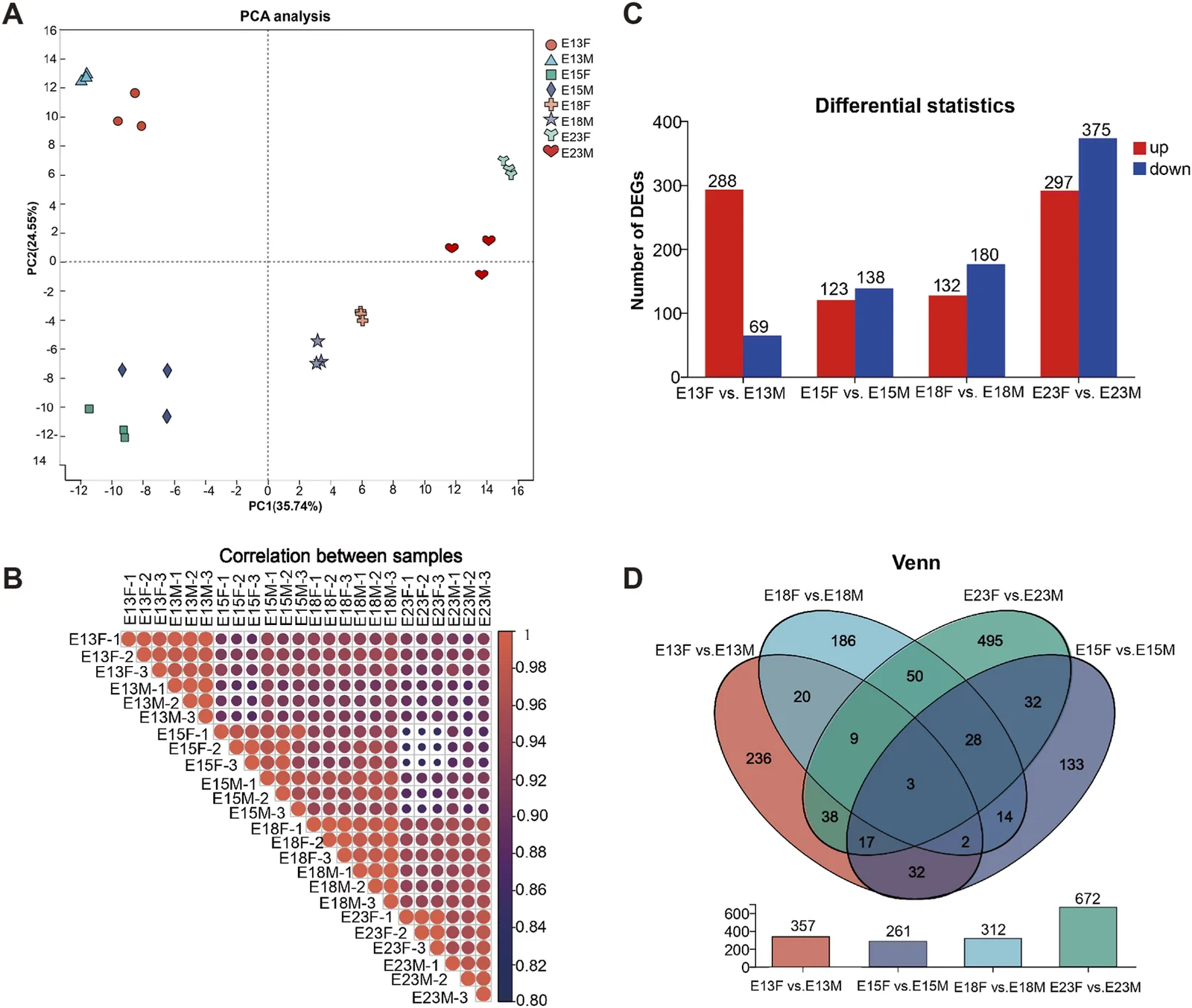
Transcriptome clustering overview and differential gene expression statistics across embryonic stages. **(A)** Principal component analysis (PCA) of RNA-seq samples from female (F) and male (M) geese at embryonic stages E13, E15, E18, and E23. PC1 (35.74% variance) and PC2 (24.55% variance) separate samples primarily by developmental stage along PC1, with additional clustering by sex along PC2. **(B)** Correlation heatmap showing overall transcriptome similarity among biological replicates and across different sample groups. **(C)** Bar charts summarizing the numbers of upregulated and downregulated differentially expressed genes (DEGs) for each pairwise comparison between females and males at E13, E15, E18, and E23 (E13F vs. E13M, E15F vs. E15M, E18F vs. E18M, and E23F vs. E23M). DEGs were defined using thresholds of adjusted p-value <0.05 and |log_2_ fold change| ≥ 1. **(D)** Venn diagram illustrating the overlaps and unique sets of sex-biased DEGs across the four embryonic stages. Numbers in each region indicate the count of DEGs specific to or shared among comparisons. DEG criteria are consistent with those in **(C)**.

Differential expression analysis was performed using thresholds of |log_2_ fold change| > 1 and adjusted P < 0.05. The numbers of differentially expressed genes (DEGs) identified between female and male samples at each stage were as follows: 357 DEGs at E13 (288 upregulated in females, 69 downregulated; Supplementary Table S2), 261 DEGs at E15 (123 upregulated in females, 138 downregulated; Supplementary Table S3), 312 DEGs at E18 (132 upregulated in females, 180 downregulated; Supplementary Table S4), and 672 DEGs at E23 (297 upregulated in females, 375 downregulated; Supplementary Table S5) (Figure 2C). Furthermore, Venn diagram analysis further revealed that these sex-related DEGs exhibit high stage specificity, with only three genes consistently showing sex-biased expression across all four developmental stages (Figure 2D). This finding indicates that sex-dependent transcriptional regulation is dynamic, transient, and highly stage-specific. Together, these data demonstrate that transcriptomic differences between female and male embryonic skeletal muscle satellite cells change dynamically throughout development, revealing the presence of sex-specific temporal regulatory programs governing embryonic muscle formation in geese.

### 3.3 Analysis of differentially expressed genes between female and male samples across four developmental stages

As shown in Figures 3A,B, heatmaps illustrate the expression dynamics of DEGs in female (F) and male (M) goose embryonic SMSCs across four developmental stages (E13, E15, E18, E23). Both sexes exhibit distinct stage-specific clustering patterns, with each time point displaying a unique gene expression profile. In females, certain gene clusters show gradually increasing or decreasing trends during development (Figure 3A). Although males also exhibit dynamic changes, their gene modules and temporal trajectories differ markedly from those in females (Figure 3B). These findings indicate that sex-dependent transcriptional programs during embryonic muscle development are highly stage-specific and exhibit distinct dynamic characteristics between sexes.

**FIGURE 3:**
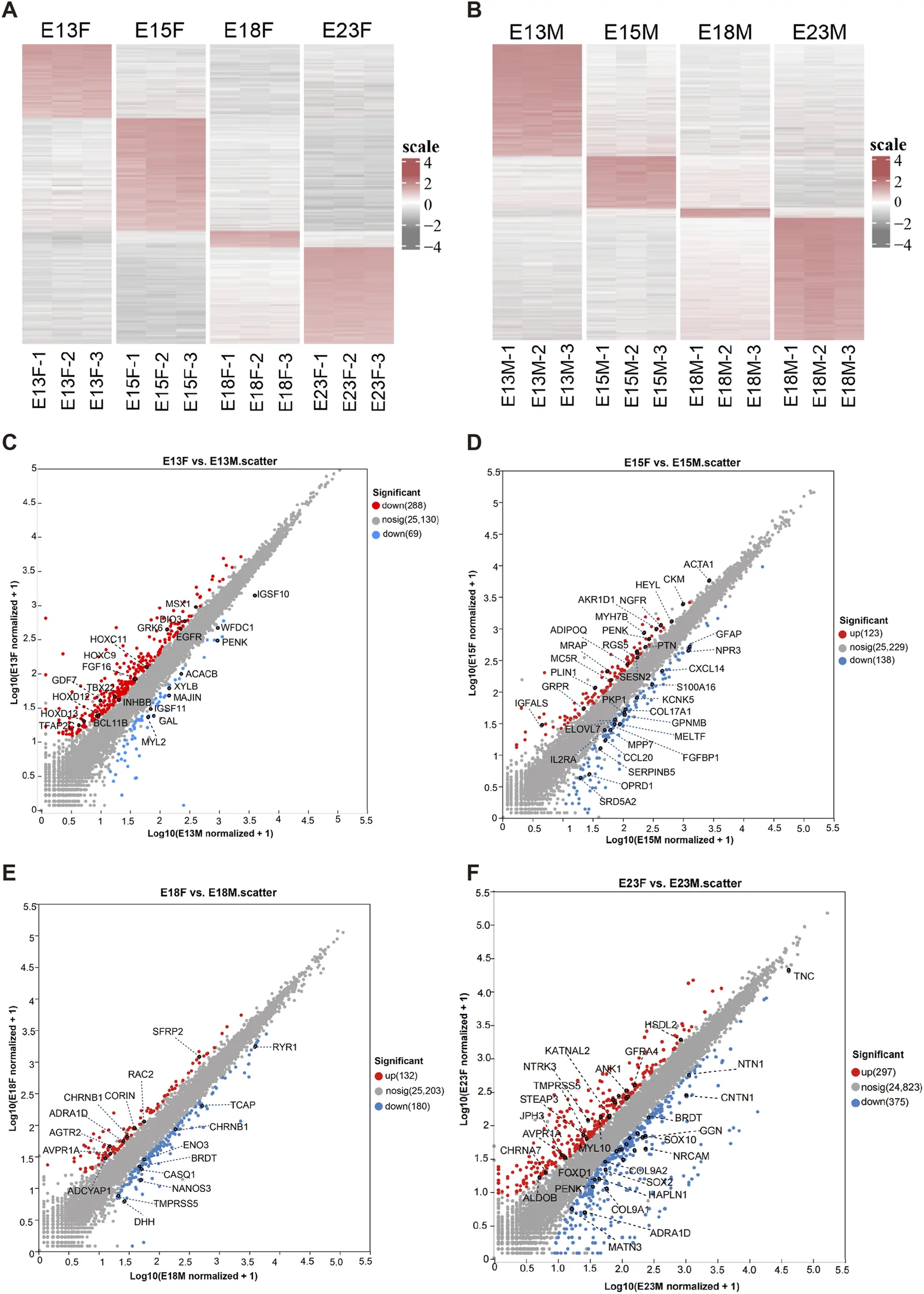
Transcriptomic profiles and identification of sex-biased differentially expressed genes (DEGs) in goose SMSCs across embryonic stages. **(A)** Expression trends in female samples at E13, E15, E18, and E23 (E13F–E23F). **(B)** Expression trends in male samples at E13, E15, E18, and E23 (E13M–E23M). **(C–F)** Scatter plots showing sex-biased DEGs at each embryonic stage: **(C)** E13F vs. E13M, **(D)** E15F vs. E15M, **(E)** E18F vs. E18M, and **(F)** E23F vs. E23M. In scatter plots, red dots represent significantly upregulated genes in females relative to males, blue dots represent significantly downregulated genes in females relative to males, and grey dots indicate genes with no significant differential expression.

To investigate potential sex-related differences in gene expression during SMSC development, we compared the transcriptomes of female and male SMSCs at the four embryonic stages. The analysis identified sets of DEGs between sexes at each time point (Figures 3C–F).

At E13, genes with higher expression in female SMSCs were associated with positional identity and development patterning (HOXC9, HOXC11, HOXD12, HOXD13, MSX1, TBX22), growth factor signaling for proliferation (GDF7, FGF16, EGFR), transcriptional/epigenetic regulation (BCL11B, TFAP2C, GRK6), and endocrine/metabolic modulation (DIO3, INHBB). Additionally, female-biased genes included HRH2, HTR2A, and TFAP2C (Figure 3C). Conversely, genes with higher expression in male SMSCs (i.e., downregulated in females) included those encoding membrane adhesion molecules (IGSF10, IGSF11, MAJIN) and markers associated with early differentiation or metabolic processes (MYL2, ACACB, WFDC1, XYLB, PENK, GAL) (Figure 3C).

At E15, genes with higher expression in female SMSCs were involved in myofiber maturation (ACTA1, CKM, MYH7B, METRN), lipid metabolism (PLIN1, ADIPOQ), developmental signaling (NGFR, PTN, HEYL, RGS5, IGFALS), steroidogenic enzymes (AKR1D1, MRAP), and neuropeptide/GPCR signaling (GRPR, MC5R, PENK). In male SMSCs at this stage, higher expression was observed for genes involved in extracellular matrix remodeling (MMP7, GPNMB, FGFBP1, COL17A1), immune-related processes (CCL20, IL2RA, CXCL14), calcium signaling (S100A16, KCNK5), structural stabilization (MELTF, GFAP, PKP1), and androgen-associated pathways (SRD5A2, ELOVL7, OPRD1, NPR3, SERPINB5), as well as SESN2 (Figure 3D).

At E18, genes with higher expression in female SMSCs were associated with signal integration and microenvironment regulation, including SFRP2, OCLN, SYNDIG1, and several neuroendocrine receptors (ADCYAP1, ADRA1D, AGTR2, AVPR1A). In male SMSCs at this stage, genes with higher expression were involved in contractile apparatus maturation (RYR1, CASQ1, TCAP, ENO3, CHRNB1), and included several testis-associated genes (BRDT, DHH, NANOS3, TMPRSS5) (Figure 3E).

At E23, gene with higher expression in female SMSCs were related to structural and metabolic processes (ANK1, JPH3, SLC4A1, ALDOB, HSDL2, MEOX1, TAL1), neuromuscular signaling (GFRA4, NTRK3, CHRNA2, CHRNA7), and hormonal microenvironment regulation (TMPRSS5, ARPP21, KATNAL2, HSD11B2, STEAP3, AVPR1A). In male SMSCs at E23, higher expression was observed for genes involved in extracellular matrix organization (COL11A2, COL8A1, COL9A1, COL9A2, MATN3, ACAN, HAPLN1), neuromuscular junction maturation (SYNDIG1, CNTN1, NRCAM, NTN1), and systemic masculinization-associated processes (BRDT, YBX2, GGN; low expression of RSPO1, SOX10, SOX2), along with neuroendocrine signaling genes (ADRA1D, PENK, MTNR1B) (Figure 3F).

In summary, stage-specific gene expression profiles from E13 to E23 reveal distinct temporal dynamics in sexually dimorphic genes between female and male goose SMSCs. These dynamic transcriptional programs suggest that sex significantly influences satellite cell development, potentially contributing to sex-specific differences in embryonic muscle formation.

### 3.4 GO enrichment analysis of DEGs between female and male SMSCs

GO enrichment analyses were performed on the sets of DEGs identified at each developmental stage: 357 DEGs at E13 (E13F vs. E13M), 261 at E15 (E15F vs. E15M), 312 at E18 (E18F vs. E18M), and 672 at E23 (E23F vs. E23M) (adjusted p < 0.05; Figure 4). The enrichment results for each stage are described below.

**FIGURE 4:**
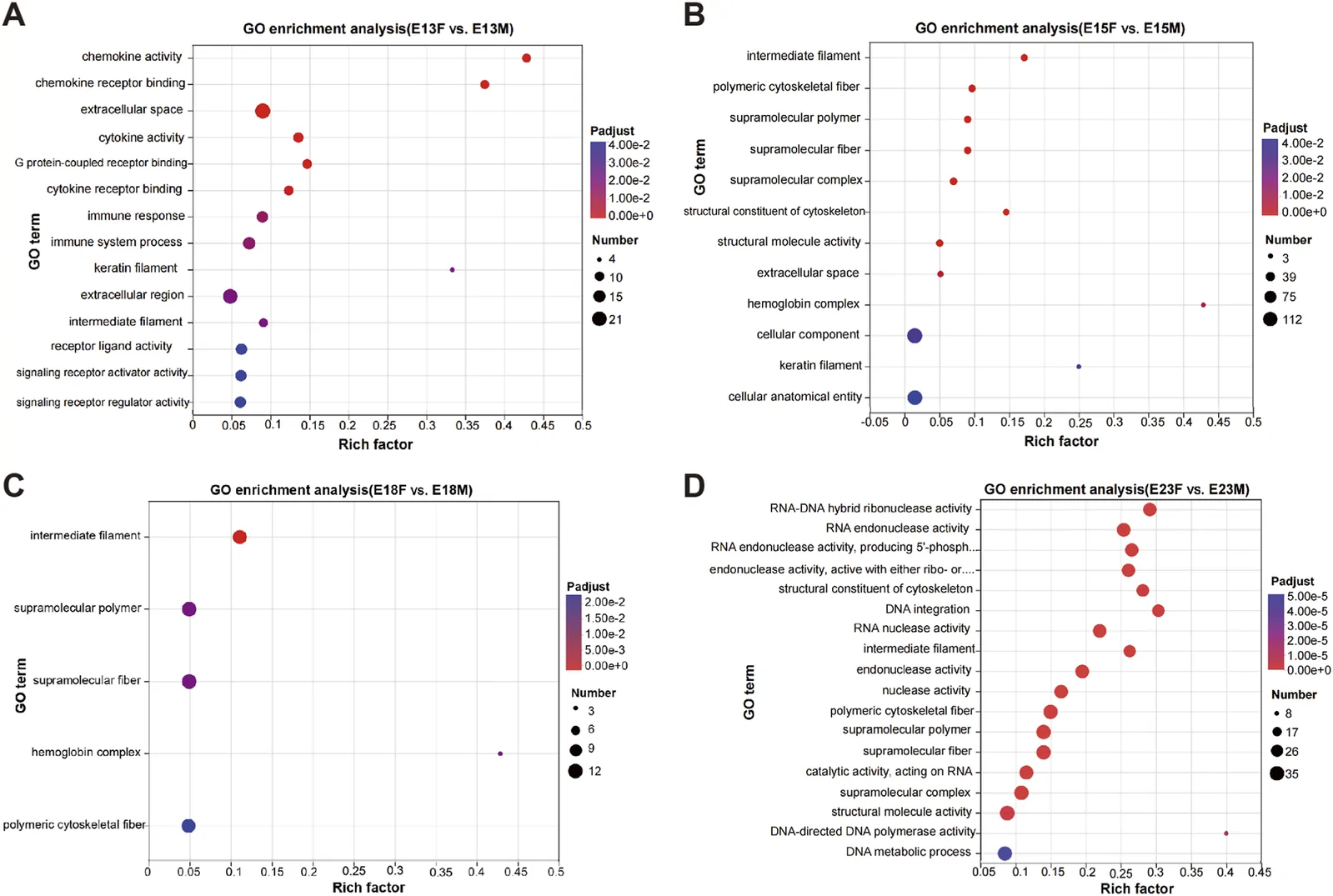
GO enrichment analysis of sex-biased DEGs across embryonic stages. **(A–D)** Enriched GO terms at E13, E15, E18, and E23, respectively. Bar color indicates adjusted P-value, and bar length represents rich factor. At E13, immune- and chemokine-related terms dominate; at E15 and E18, cytoskeletal and structural terms are enriched; at E23, nuclease and DNA metabolic terms emerge. These stage-specific GO terms reflect distinct sex-dimorphic biological processes in goose SMSCs during development.

At E13 (Figure 4A), DEGs were primarily enriched in biological processes related to immune response and immune system process. Molecular function terms included chemokine and cytokine activity, G protein-coupled receptor binding, and receptor-ligand activity. Cellular component terms were dominated by extracellular space, keratin filament, and intermediate filament. These findings suggest that early sex differences are associated with the regulation of the immune microenvironment, chemokine/cytokine signaling, and extracellular matrix interactions.

At E15 (Figure 4B), the enrichment landscape shifted notably. While immune-related terms such as immunoglobulin complex and antigen binding persisted, the dominant enriched terms included polymeric cytoskeletal fiber, supramolecular polymer/fiber/complex, structural constituent of cytoskeleton, and structural molecule activity. This transition indicates that sex differences at this stage begin to emphasize cellular structural support and cytoskeletal organization alongside sustained immune-related functions.

At E18 (Figure 4C), only cellular component terms were enriched, including intermediate filament, supramolecular polymer/fiber, hemoglobin complex, and polymeric cytoskeletal fiber, with no enrichment in biological processes or molecular functions. This implies that sex differences at this stage are mainly focused on the maintenance of cytoskeletal and supramolecular structures.

At E23 (Figure 4D), in addition to the sustained enrichment of cytoskeleton- and supramolecule-related components (supramolecular complex, supramolecular polymer, collagen trimer), newly enriched terms included DNA integration, DNA metabolic process, RNA-DNA hybrid ribonuclease activity, RNA endonuclease activity, DNA-directed DNA polymerase activity, and various nuclease activities. Additionally, terms related to chemokine activity, receptor-ligand activity, and antigen processing/presentation also appeared. These results suggest that late-stage sex differences involve DNA replication, repair, nucleic acid metabolism, while still retaining immune and structural functions.

In summary, the GO functional characteristics display a stage-dependent progression from immune regulation to structural maintenance and then to nucleic acid metabolism, while immune-related functions persist throughout development, reflecting multi-level, sex-specific regulation of skeletal muscle development.

### 3.5 KEGG enrichment analysis of DEGs between female and male SMSCs

KEGG pathway enrichment analysis (adjusted p < 0.05) was performed on the sets of DEGs identified at four developmental stages (E13, E15, E18, E23) in female and male goose embryo skeletal muscle satellite cells. The results for each stage are summarized below.

At E13 (Figure 5A), DEGs were significantly enriched in multiple pathways (adjusted p < 0.05). The most prominently enriched pathways were those related to immune and inflammatory responses, including the IL-17, Toll-like receptor, NF-κB, chemokine, and cytokine-cytokine receptor interaction pathways. In addition, significant enrichment was observed in the cell adhesion and ECM-receptor interaction pathway, the estrogen signaling pathway, and the PI3K-Akt signaling pathway. These findings indicate that early sex differences primarily involve immune regulation, inflammatory responses, cell-matrix interactions, and hormone signaling.

**FIGURE 5:**
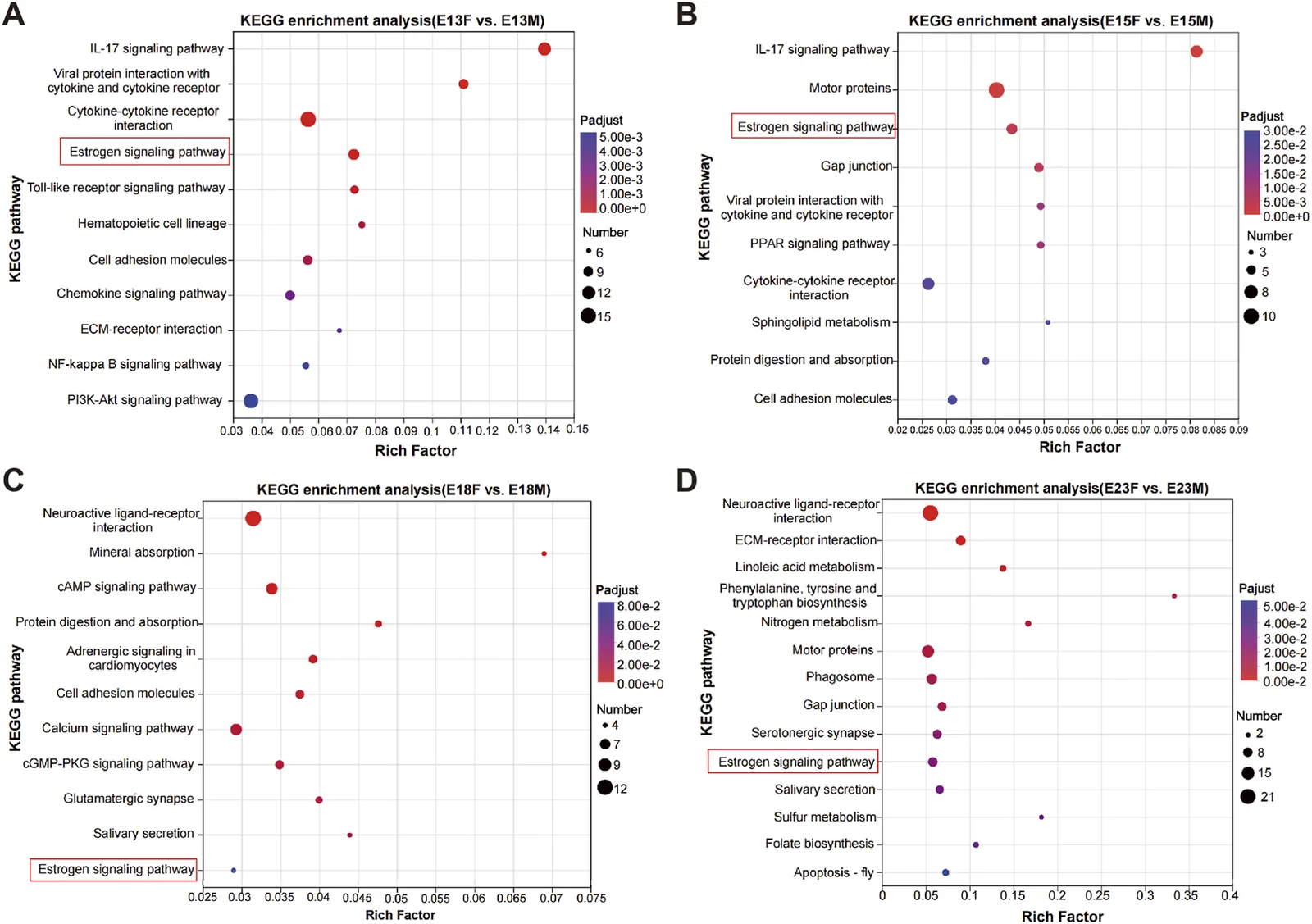
KEGG enrichment analysis of sex-biased DEGs at four embryonic stages. **(A–D)** Enriched KEGG pathways at E13, E15, E18, and E23, respectively. Bar color indicates adjusted *p*-value, and bar length represents rich factor. Key pathways include cytokine-cytokine receptor interaction, ECM-receptor interaction, estrogen signalling, and neuroactive ligand-receptor interaction, revealing stage-specific sex-dimorphic signalling in goose SMSCs.

At E15 (Figure 5B), significantly enriched pathways included immune- and inflammation-related terms (IL-17, viral protein interaction with cytokine and cytokine receptor, cytokine-cytokine receptor interaction), cytoskeleton and motility-related terms (motor proteins, gap junction), hormone signaling and metabolism (estrogen signaling, PPAR signaling, sphingolipid metabolism), as well as cell adhesion and matrix interaction. Compared with E13, newly emerged pathways at E15 included motor proteins, gap junction, PPAR signaling, and sphingolipid metabolism, suggesting a shift from immune-dominated regulation toward cell motility, metabolic reprogramming, and intercellular communication.

At E18 (Figure 5C), significantly enriched pathways included signal transduction (cAMP, calcium, cGMP-PKG, neuroactive ligand-receptor interaction, adrenergic signaling), cell adhesion and matrix interaction, mineral absorption, glutamatergic synapse, and secretion (salivary secretion). The estrogen signaling pathway showed a near-significant trend (adjusted p < 0.07). Notably, immune- and inflammation-related pathways were not enriched at this stage, indicating a transition toward the reprogramming of intracellular signal transduction and the establishment of neuron-like interaction features.

At E23 (Figure 5D), significantly enriched pathways covered signal transduction (neuroactive ligand-receptor interaction, estrogen signaling, serotonergic synapse), cytoskeleton and motility, ECM-receptor interaction, metabolism-related pathways (linoleic acid metabolism; phenylalanine, tyrosine and tryptophan biosynthesis; nitrogen metabolism; sulfur metabolism; folate biosynthesis), phagosome and apoptosis (apoptosis-fly), as well as secretion (salivary secretion). Compared with earlier stages, E23 exhibited pronounced metabolic reprogramming characteristics, along with the emergence of phagosome and apoptosis-related pathways, suggesting that late-stage sex differences involve metabolic adaptation, cellular clearance mechanisms, and extracellular matrix remodeling.

In summary, the KEGG pathway enrichment profiles of male and female goose embryo SMSCs display a stage-dependent evolution: from immune/inflammation and hormone signaling dominance at E13, to cell motility and metabolic reprogramming at E15, followed by signal transduction reprogramming at E18, and finally a complex regulatory pattern integrating metabolism, phagocytosis, and apoptosis at E23.

### 3.6 Protein–protein interaction (PPI) analysis

To investigate functional interactions among DEGs and identify potential hub genes underlying sex-dimorphic development of goose SMSCs, PPI networks were constructed for each developmental stage (E13, E15, E18, and E23) using the STRING database and visualized in Cytoscape (Figures 6A–D).

**FIGURE 6:**
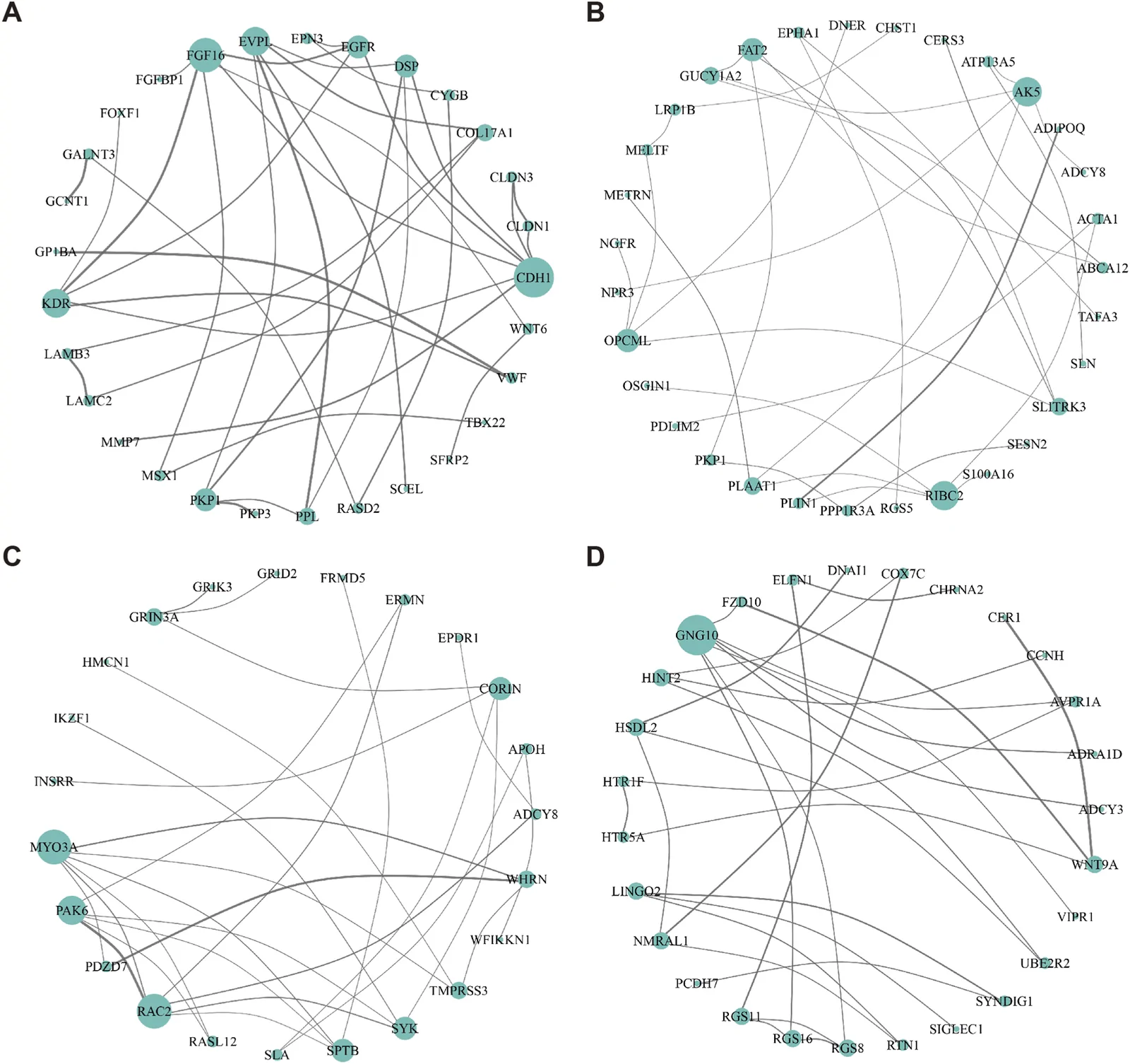
Protein-protein interaction (PPI) networks of differentially expressed genes (DEGs) in goose skeletal muscle satellite cells (SMSCs) during sex-dimorphic development (E13–E23). **(A–D)** PPI networks at embryonic days E13, E15, E18, and E23, respectively. The networks were constructed using the STRING database and visualized with Cytoscape. Each node represents a protein encoded by a DEG, and each edge denotes a predicted functional interaction between two proteins.

At E13 (Figure 6A), the PPI network centers on EGFR, which connects to FGF16, KDR, MMP7, and CLDN1, linking growth factor signaling, angiogenesis, and extracellular matrix (ECM) remodeling. A module comprising cell junction and desmosome proteins (CDH1, CLDN1/3, DSP, EVPL, PKP1/3, PPL) underscores sex-biased differences in cell adhesion architecture. Furthermore, an ECM-related subnetwork (COL17A1, LAMB3, LAMC2, VWF) engages with MMP7 and EGFR, implicating sex-dimorphic regulation of cell–cell communication, matrix homeostasis, and early vascularization.

At E15 (Figure 6B), the PPI network is characterized by hub genes including ACTA1, ADIPOQ, PKP1, and SESN2. The network highlights modules involved in myofiber maturation (ACTA1), lipid metabolism (ADIPOQ, PLIN1), cell adhesion (PKP1), and stress response (SESN2). These interactions suggest that sex differences at E15 are primarily associated with cytoskeletal organization, metabolic regulation, and cellular structural integrity.

At E18 (Figure 6C), the PPI network identifies RAC2 as a key hub. RAC2 shows strong interactions with multiple genes, including MYO3A, PAK6, and SPTB. These tightly interconnected genes form a core module, suggesting that the network is primarily involved in cytoskeletal reorganization, cell motility, and morphological maintenance.

At E23 (Figure 6D), GNG10 serves as a central hub, around which multiple functional gene clusters are organized. The interconnection of WNT9A with FZD10, ADRA1D, and ADCY3 suggests involvement of the Wnt signaling pathway. At the periphery, RGS11, RGS16, and RGS8 (regulators of G-protein signaling) are interconnected and linked to the main network. The network also includes GPCR-related genes such as HTR1F, HTR5A, and AVPR1A. Collectively, these findings indicate that DEGs between E23F and E23M are primarily involved in G-protein signaling, the Wnt pathway, and receptor regulation.

In summary, the stage-specific PPI networks (E13–E23) demonstrate a dynamic shift in sex-dimorphic functional modules: from EGFR-mediated signaling and ECM remodeling at E13, to cytoskeletal and metabolic regulation at E15, to RAC2-driven cytoskeletal reorganization at E18, and finally to GNG10-centered G-protein and Wnt signaling at E23.

### 3.7 Validation of DEGs by qPCR

To validate the RNA-seq results, we conducted qPCR analysis on eight randomly selected DEGs across four developmental stages (E13, E15, E18, and E23). As shown in Figures 7A–H, the expression patterns and sex-biased directions of FGF16, IGSF10, ACTA1, MMP7, GATA6, BRDT, ANK1, and YBX2 were strongly corroborated by qPCR. This high degree of concordance between the two methodologies unequivocally supports the reliability of the transcriptomic dataset.

**FIGURE 7:**
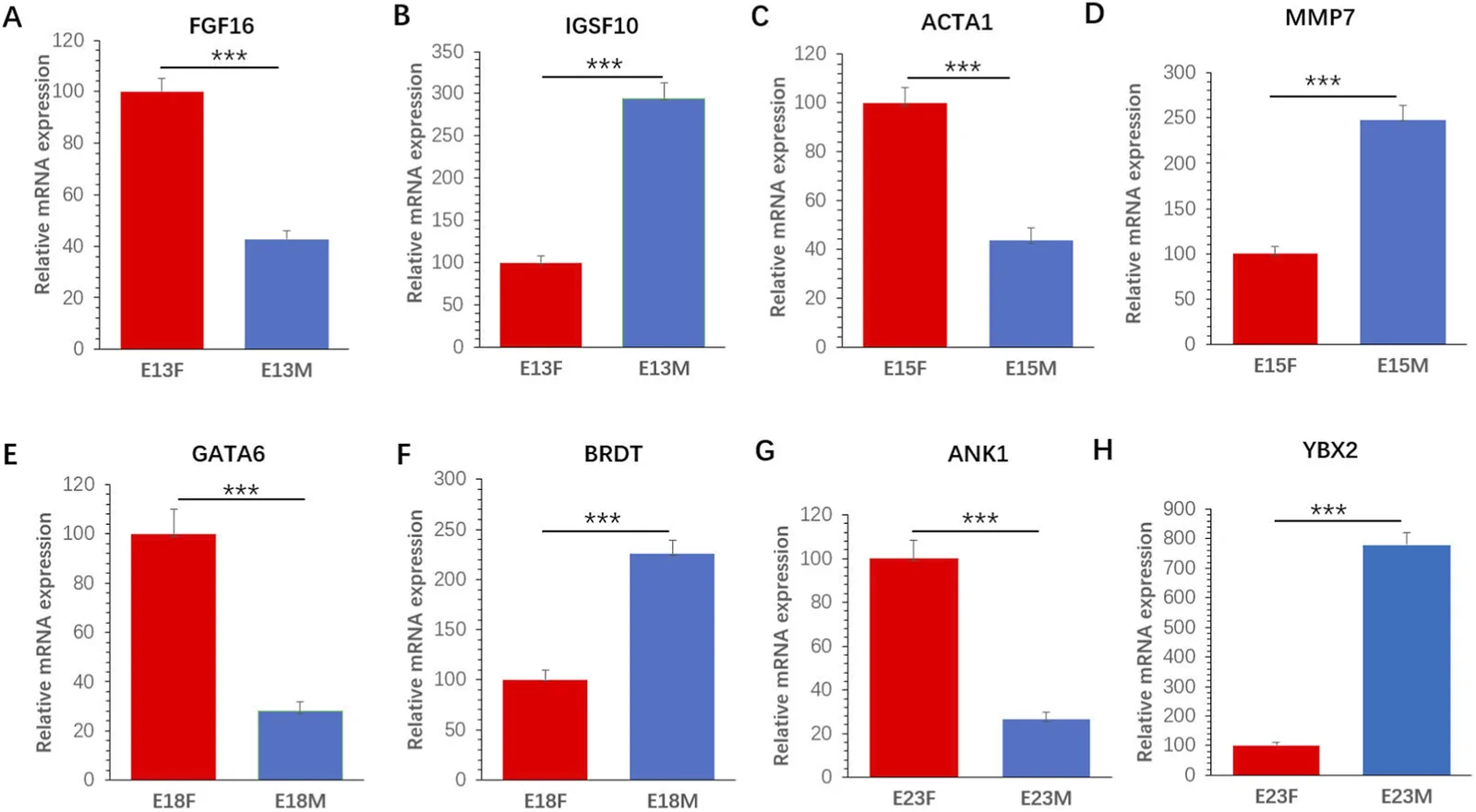
qPCR validation of key DEGs involved in muscle development. The mRNA levels of eight genes—**(A)** FGF16, **(B)** IGSF10, **(C)** ACTA1, **(D)** MMP7, **(E)** GATA6, **(F)** BRDT, **(G)** ANK1, and **(H)** YBX2—were examined in SMSCs derived from leg muscles of female and male ZW geese at E13, E15, E18, and E23. Values represent mean ± SEM (n = 3 per stage). ***p < 0.001 indicates statistically significant differences.

## 4 Discussion

Sexual dimorphism in skeletal muscle development is critical for poultry breeding. Traditional studies have focused on post-hatch sex differences driven by sex hormones (Henry and Burke, 1998; Yang, et al., 2024). In chickens, male embryos exhibit a great number of smaller myofibers than females, predisposing them to greater post-hatch growth (Henry and Burke, 1998). Our previous study in 10-week-old geese revealed systemic sex effects on muscle gene expression (Yang et al., 2024). However, sex differences in the embryonic SMSC pool remain unexplored. In the present study, leg muscle histology from E13 to E23 was similar between sexes, but PAX7^+^ satellite cell number and density showed significant sexual dimorphism: males exceeded females at E18, with the pattern reversing at E23. This reversal coincides with the onset of rapid leg muscle growth (E23), suggesting that sex-specific SMSC expansion may underlie post-hatch growth differences (Weng et al., 2025). Our findings extend sexual dimorphism research to mid-late embryogenesis, challenging the view that embryonic muscle development is sexually indifferent.

Transcriptomic technologies have been widely used to uncover stage-specific regulatory mechanisms of muscle development in poultry. Shi et al. (2022) identified stage-specific activation of Wnt, Notch, and MAPK pathways in embryonic chicken leg muscles using miRNA sequencing. Shao et al. (2025) reported that Shitou geese exhibit superior embryonic leg muscle development compared to Wuzong geese, with higher enrichment of muscle-related pathways and a more distinct gene expression profile. We previously constructed a developmental transcriptomic atlas of goose SMSCs from E15 to E23 and observed a transition from myofibrillar gene expression toward lipid metabolism and ECM remodeling (Wang et al., 2026b). However, these studies focused primarily on single sex or inter-breed differences, leaving the molecular basis of sexual dimorphism at the SMSC level unexplored. In the present study, we identified a large number of sex-biased and highly stage-specific DEGs in goose embryonic SMSCs (357 at E13, 261 at E15, 312 at E18, and 672 at E23). Notably, only three genes were consistently differentially expressed across all four stages, indicating that sex-dependent transcriptional regulation is highly dynamic and transient. Specifically, at E13, female-biased genes included positional identity genes (HOXC9/11, HOXD12/13) and proliferation-related signaling molecules (EGFR, FGF16); at E15, male-biased genes involved ECM remodeling (MMP7, COL17A1) and androgen-associated genes (SRD5A2); at E18, male-biased genes were associated with contractile apparatus maturation (RYR1, CASQ1); and at E23, pronounced metabolic and neuromuscular divergence emerged, with females highly expressing structural and metabolic genes (ANK1, JPH3), and males highly expressing collagen genes and systemic masculinization-associated genes (BRDT, YBX2). These sex-biased gene sets are markedly distinct from those identified in adult geese (e.g., IGF1, GHRHR, NCAPG-LCORL) (Yang et al., 2024), suggesting that the transcriptional differences observed in embryonic SMSCs precede post-hatch hormonal surges and may lay the foundation for muscle sexual dimorphism. However, given that the SMSCs were isolated from embryonic tissues that are exposed to endogenous hormonal microenvironments, we cannot exclude the potential influence of embryonic sex hormones. Therefore, we cautiously conclude that these early transcriptional programs are established prior to post-hatch endocrine changes and likely represent an initial layer of sexually dimorphic regulation in muscle development, rather than definitive evidence of strictly cell-autonomous control.

Meanwhile, GO and KEGG enrichment analyses revealed a progressive shift in functional themes underlying sex differences during embryonic development. At E13, DEGs were enriched in immune-related pathways (e.g., IL-17, Toll-like receptor, NF-κB), along with estrogen and PI3K-Akt signaling, suggesting that early sex differences may regulate SMSC proliferation and migration by shaping the immune microenvironment. At E15, enrichment shifted toward cytoskeletal polymerization, motor proteins, PPAR signaling, and sphingolipid metabolism, marking a transition from immune dominance to structural support and metabolic reprogramming. At E18, immune-related enrichment was no longer observed, whereas cAMP, calcium signaling, and neuroactive ligand-receptor interaction pathways became prominent, indicating a focus on intracellular signal transduction and neuromuscular interaction establishment. At E23, enriched pathways expanded to include DNA replication/repair, nucleic acid metabolism, phagosome, apoptosis, and various amino acid and lipid metabolic pathways, accompanied by persistent ECM-receptor interaction activation. This stage-dependent evolution (“immune → structural → signaling → metabolic”) is partially conserved with pathway dynamics observed in embryonic chicken muscles (Shi et al., 2022), but the present study links these dynamics to the sex dimension for the first time, revealing differential metabolic preparation of male and female SMSCs to meet distinct post-hatch growth demands. Protein-protein interaction (PPI) network analyses further supported this hierarchical progression, with hub genes transitioning from EGFR (E13) to ACTA1/ADIPOQ (E15) to RAC2 (E18) to GNG10 (E23), providing systems biology evidence for the multi-layered regulation of sexual dimorphism.

Several limitations of this study should be acknowledged. First, the investigation of SMSCs was restricted to the embryonic period (E13–E23), which precludes establishing a direct causal link between sex-biased properties of the embryonic satellite cell pool and post-hatch muscle hypertrophy or body weight dimorphism (Weng et al., 2025). Second, this study focused exclusively on leg muscle SMSCs. Given the asynchronous developmental dynamics between goose breast and leg muscles, as well as the recognized heterogeneity of satellite cells in breast muscle (Velleman, 2022), cross-tissue comparative studies are warranted. To comprehensively address this heterogeneity, single-cell RNA sequencing (scRNA-seq) represents a necessary next step to resolve SMSC subpopulation compositions and their distinct contributions to myogenesis. Third, our current findings rely predominantly on bulk transcriptomic profiling and lack functional validation of candidate DEGs or hub genes (e.g., BRDT, GNG10, RAC2). Additionally, while we quantified satellite cell abundance via PAX7 immunofluorescence, we did not cross-reference these protein-level data with PAX7 mRNA expression from the RNA-seq dataset. Future studies should therefore include such a transcript–protein comparison, ideally complemented by proteomic profiling, to clarify whether the observed sex- and stage-related differences in satellite cell pool size are governed at the transcriptional level or through post-transcriptional/post-translational mechanisms. Overall, subsequent work should establish a continuous embryonic-to-post-hatch developmental model, integrate single-cell multi-omics (scRNA-seq and ATAC-seq) with hormone intervention and CRISPR-Cas9-mediate gene editing for functional validation, and extend comparative analyses across breast and leg muscle tissues. Such efforts will facilitate the translation of embryonic sex differences into practical breeding applications, including early sex identification and sex-specific nutritional programming.

## 5 Conclusion

In summary, despite convergent histological outcomes between sexes, the embryonic SMSC pool in geese exhibits profound sex- and stage-specific transcriptional dynamics. From E13 to E23, sex-biased DEGs progressively shift from immune-related and positional identity genes toward metabolic, contractile, and ECM-related programs. Within this temporal trajectory, stage-specific hub genes (EGFR, ACTA1/ADIPOQ, RAC2, GNG10) being prominently associated with these temporal transitions. These findings suggest that sexual dimorphism in avain skeletal muscle may have transcriptional origins at the embryonic satellite cell stage, providing a nuanced perspective that complements the conventional view emphasizing post-hatch dominance. Collectively, this study established a comprehensive transcriptomic association resource, which may serve as a valuable reference for hypothesis generation in future functional studies and for design of sex-specific poultry breeding strategies.

## Data Availability

The datasets presented in this study can be found in online repositories. The names of the repository/repositories and accession number(s) can be found in the article/Supplementary Material.

## Supplementary material

Supplementary Table S1 Raw quantitative cell-count data.

Supplementary Table S2 DEGs in E13F vs. E13M comparison.

Supplementary Table S3 DEGs in E15F vs. E15M comparison.

Supplementary Table S4 DEGs in E18F vs. E18M comparison.

Supplementary Table S5 DEGs in E23F vs. E23M comparison.
